# The supporting role of Schwann cells in perineural invasion of pancreatic ductal adenocarcinoma

**DOI:** 10.3389/fphar.2025.1540027

**Published:** 2025-06-11

**Authors:** Yun He, Zheng Chen, Liu Yang, Shuanying Qiao, Zonghua Su, Feng Ding, Fadian Ding, Fuli Xin, Siyu Xiang, Aiping Lyu, Fangfei Li

**Affiliations:** ^1^ Shum Yiu Foon Shum Bik Chuen Memorial Centre for Cancer and Inflammation Research, School of Chinese Medicine, Hong Kong Baptist University, Kowloon, Hong Kong SAR, China; ^2^ School of Chinese Medicine, Institute of Precision Medicine and Innovative Drug Discovery (PMID), Hong Kong Baptist University, Kowloon, Hong Kong SAR, China; ^3^ School of ChineseMedicine, Law Sau Fai Institute for Advancing Translational Medicine in Bone and Joint Diseases, Hong Kong Baptist University, Kowloon, Hong Kong SAR, China; ^4^ Department of Hepatobiliary and pancreatic Surgery, The first Affiliated Hospital, Fujian Medical University, Fuzhou, China; ^5^ Department of Hepatobiliary and Pancreatic Tumor Surgery, Fujian Medical University Cancer Hospital, Fujian Cancer Hospital, Fuzhou, China

**Keywords:** pancreatic ductal adenocarcinoma, perineural invasion, Schwann cells, tumor microenvironment, peripheral nervous system

## Abstract

Pancreatic ductal adenocarcinoma (PDAC) is a highly aggressive cancer, with tumor cells readily disseminating to other organs through the bloodstream, lymphatic system, and nervous system, thereby impacting patients’ survival rates. PDAC is often associated with perineural invasion (PNI), which not only facilitates tumor spread but may also lead to symptoms such as pain, further affecting the patient’s quality of life. PNI is frequently observed in PDAC and has become an important histopathological marker associated with poor clinical outcomes. Many studies suggest that a high density of Schwann cells (SCs) is typically found in areas of PNI in PDAC. What’s more, as the primary glial cells in the PNS, SCs actively contribute to pancreatic tumour progression by releasing substances capable of interacting with cancer cells and promoting cancer cells proliferation and migration in tumor microenvironment (TME). Therefore, SCs are crucial in the interactions between nerves and tumors as the primary glial cells within PNS. In this review, our objective is to present novel insights and perspectives for PDAC therapy that targets SCs and related signal pathways to decrease PNI, thereby reduce pain and prolong survival in cancer patients. We detail and summarize the multiple mechanisms by which SCs promote PNI in tumors and thus lead to malignancy.

## 1 Introduction

Pancreatic ductal adenocarcinoma (PDAC) is often diagnosed at an advanced metastatic stage, characterized by its aggressive nature and poor prognosis. In the advanced stages of PDAC, metastasis primarily occurs through three main pathways: lymphatic, neural, and hematogenous (vascular) dissemination (Avula et al., 2020). The sympathetic and parasympathetic nerves, which are part of the peripheral nervous system (PNS), play a role in controlling the pancreas and pancreatic cancer cells tend to invade the nerve bundles within the pancreas. Therefore, a representative feature of PDAC is neuropathy, primarily manifested by PNI, the infiltration of cancer cells along neural pathways promotes a series of biochemical and physical interactions that can stimulate both axonal sprouting, tumor cell proliferation and provide additional pathways for cancer to spread (Kanda et al., 2012; Mehta et al., 2022). PNI also leads to nerve-related abdominal pain in patients and is a major contributor to the pain associated with PDAC (Liebl et al., 2014). PNI is recognized as an independent factor that predicts poor prognosis in patients diagnosed with PDAC (Zhang et al., 2013). Consequently, PNI significantly contributes to the pathophysiology of PDAC, profoundly affecting both tumor aggression and cancer-associated pain (Tu et al., 2023). The current understanding of the mechanisms underlying PNI is insufficient and effective treatment options for PDAC are scarce. This situation highlights the urgent need for further research to unravel the biological processes involved in PDAC development and progression, and to develop more effective therapeutic strategies (Grigorescu et al., 2024; Ho et al., 2020; Malikowski et al., 2020; Adamska et al., 2017).

The primary glial cells in the PNS, known as SCs, originated from the neural crest. SCs generally exist as immature cells but have the capacity to differentiate into a range of cell types, including odontoblasts, melanocytes, autonomic neurons, and enteric glia, depending on the conditions (Milichko and Dyachuk, 2020). Subsequently, the immature SCs differentiate into myelinating or non-myelinating SCs (Taveggia and Feltri, 2022; Sinegubov et al., 2022). SCs demonstrate a strong affinity for cancer cells and initiate interactions between nerves and cancer cells. These interactions play a significant role in critical cancer processes, such as tumor migration, invasion, immune evasion, and the transmission of cancer-related pain, including that associated with bone cancers and breast cancers (Zhang et al., 2023; Hao et al., 2023). However, studies of SCs in PDAC remain relatively scarce. Furthermore, despite the aggressive nature of PDAC and the recognised role of nerve-tumor interactions in its progression, a systematic review summarising the current knowledge of SCs contributions to PNI in PDAC is lacking.

In this review, we provide an overview of past research findings and explore the present knowledge concerning PDAC neuropathy. We summarized the cellular and molecular mechanisms by which SCs facilitate PNI and induce pain in PDAC. Additionally, it’s important for future research directions for improving the diagnosis, prognosis, and treatment of this highly aggressive and dreaded disease.

## 2 Perineural invasion in pancreatic ductal adenocarcinoma

### 2.1 Intrapancreatic innervation

The pancreas is regulated by a network of sensory branches along with sympathetic and parasympathetic nerve fibers. This network functions as a conduit for transmitting sensations from the pancreas and conveying additional sensory information (Ding et al., 2024). Researchers believed that the sympathetic innervation of the pancreas originates from the dorsal root ganglia, and sympathetic sends nerve impulses to the pancreas via the pancreatic plexus, which is involved in functions such as regulation of exocrine and endocrine secretion, blood flow regulation, sensation and pain in the pancreas (Li W. et al., 2019; Nicoletti et al., 2024). The balance between sympathetic and parasympathetic is crucial for running normal physiological function of the pancreas, and disturbances in this neuromodulation can lead to pancreatic diseases, including PDAC (Kiba, 2004). The development of chronic pancreatitis and pancreatic cancer in humans, intrapancreatic nerves undergo hypertrophy, increasing in size, and exhibit a higher density, with a growing number of nerve fibers (Demir et al., 2015). A study comparing nerve fibres in 256 patients with PDAC, CP and PDAC patients had significant hypertrophy of interlobular nerve fibres compared with who had normal pancreas, and a statistically significant association was observed between elevated neuroinvasion and reduced overall survival (OS) in patients with PDAC (Iwasaki et al., 2019). Pancreatic neuropathy has positive correlation with neuropathic pain by analyzing pancreatic pathologies in 546 patients with chronic pancreatitis (CP) and PDAC (Ceyhan et al., 2009). Current research suggests that nerves might play a crucial role in the dissemination of PDAC (Li et al., 2021).

### 2.2 Clinical epidemiology of PNI in PDAC

**FIGURE 1:**
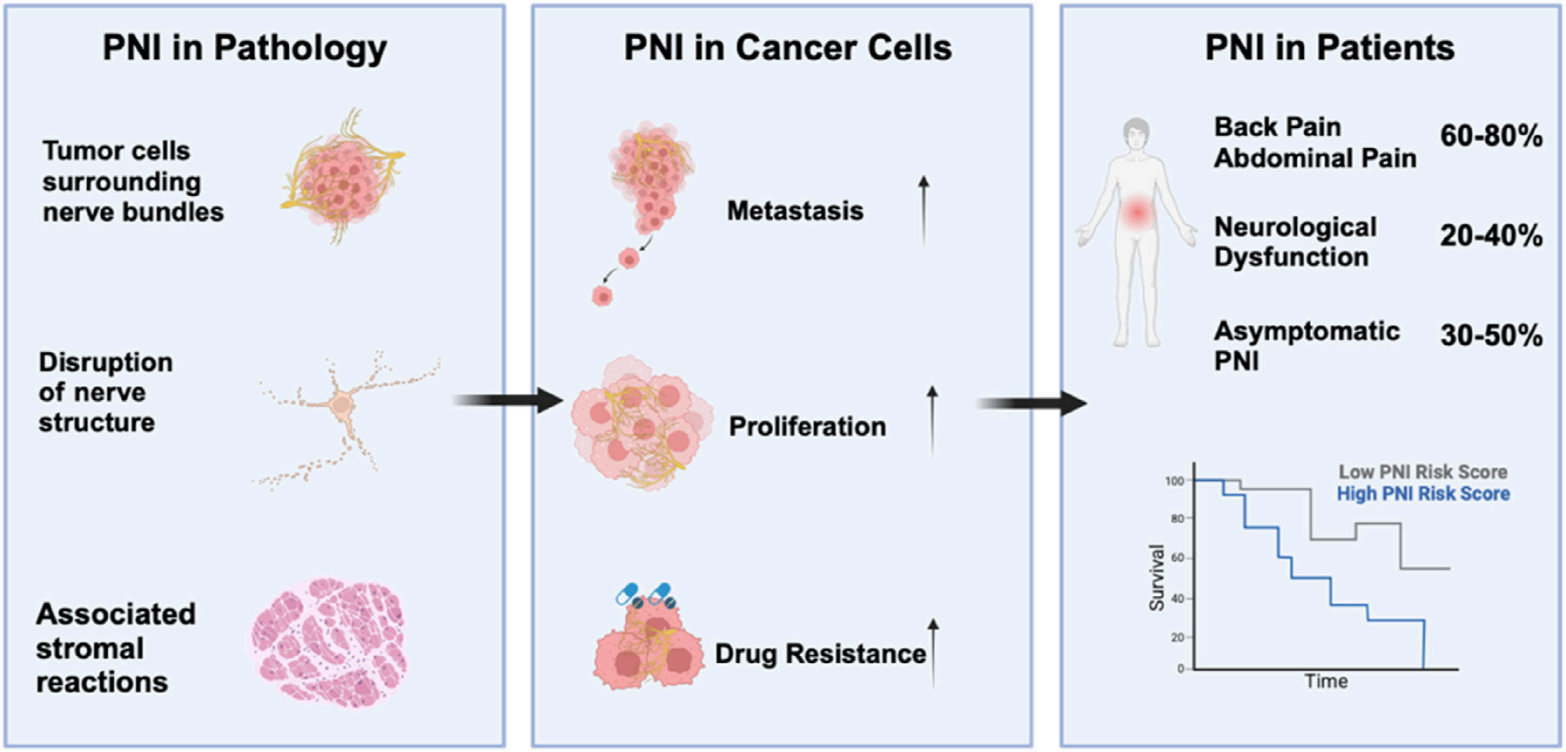
Pathological and Clinical Implications of Perineural Invasion (PNI) in PDAC: This figure illustrates the pathological features of PDAC. Perineural invasion promotes metastasis and proliferation in cancer cells and also regulates drug resistance. Pancreatic ductal adenocarcinoma patients are more likely to experience neuropathic symptoms such as back, abdominal pain, neurological dysfunction and asymptomatic PNI. Additionally, high perineural invasion infiltration in patients is associated with a poor prognosis (Zhang et al., 2013).

According to several clinical studies, increasing the severity of PNI reduced survival in PDAC patients exclusively (Selvaggi et al., 2022). Tissues from 422 patients with tumor invasion of the nerve plexus were analysed, and it was found that cancer cells were present in the nerve space, and that PNI significantly affected the prognosis of patients on multivariate analysis. Of the 109 patients with PDAC, 75 (68.8%) were positive for nerve infiltration (Tan et al., 2021). Multivariate COX regression analysis revealed a correlation between PNI and lymph node metastasis, pancreatitis, and CA19-9 levels (P < 0.05). Additionally, PNI has been recognized as a distinct indicator of poor prognosis in pancreatic cancer (P < 0.05) (Yang et al., 2017). Other studies also found that tumor invasion of peripheral nerves was present in 531 (93%) of 571 patients who underwent surgical resection for therapeutic PDAC, with the majority of PNI-positive patients showing advanced tumor and lymph node invasion (Felsenstein et al., 2022). In conclusion, PNI is typically linked to more aggressive tumors, elevated recurrence rates, and reduced survival outcomes (Figure 1). Therefore, there is a greater need to improve our understanding and research into PNI and related mechanisms in PDAC to pursue therapeutic strategies.

### 2.3 *In vitro* and *in vivo* models for PNI in PDAC

Research on PNI in PDAC relies on various *in vitro* and *in vivo* models. However, the limitations of these models significantly hinder a deeper understanding of the mechanisms underlying PNI. The simplifications inherent in *in vitro* models further restrict mechanistic exploration. Neural-cancer cell co-culture systems, such as dorsal root ganglia co-cultured with PDAC cells, or three-dimensional matrix models can simulate chemotactic migration, they lack the complexity of multi-component interactions, including blood vessels and immune cells, which are essential for capturing the intricacies of the tumor-nerve microenvironment (Ayala et al., 2001; Ceyhan et al., 2008). Additionally, molecular intervention studies based on Schwann cell conditioned media can identify the roles of neurotrophic factors, but they overlook the regulatory effects of physical cell-to-cell contact and mechanical signals within the microenvironment (Pettingill et al., 2008).

*In vivo* models, such as the in pancreatic tumor model, simulate the natural disease progression by injecting PDAC cells into the mouse pancreas. Although this approach preserves the tumor microenvironment, including interactions between nerves and stromal cells, PNI formation is slowand exhibits significant individual variability, making it challenging to dynamically track real-time interactions between cancer cells and SCs. The sciatic nerve invasion model accelerates PNI formation through ectopic injection, allowing for quantification of cancer cell migration along the nerve; however, it fails to replicate the specific local pancreatic microenvironment, leading to a disconnect in mechanistic studies (Deborde et al., 2018). KPC mouse models can spontaneously develop PDAC accompanied by PNI, but the multi-step carcinogenic process and the differing time scales compared to human disease may obscure key early driving events of PNI (Huang et al., 2023; Hirth et al., 2022). Collectively, these model limitations result in a fragmented understanding of PNI mechanisms: *in vivo* models struggle to elucidate the dynamic interactions between SCs and cancer cells in real time, while *in vitro* systems overly simplify the microenvironment, failing to reveal the multidimensional synergistic effects of immune suppression, nerve remodeling, and matrix stiffening. Future efforts should integrate high-resolution *in vivo* imaging, multi-component organ co-culture systems, and single-cell spatiotemporal omics to overcome existing bottlenecks and comprehensively decode the molecular and cellular driving networks of PNI.

## 3 Function of Schwann cells in perineural invasion of pancreatic ductal adenocarcinoma

### 3.1 SCs: the primary glial cells within the PNS

**FIGURE 2:**
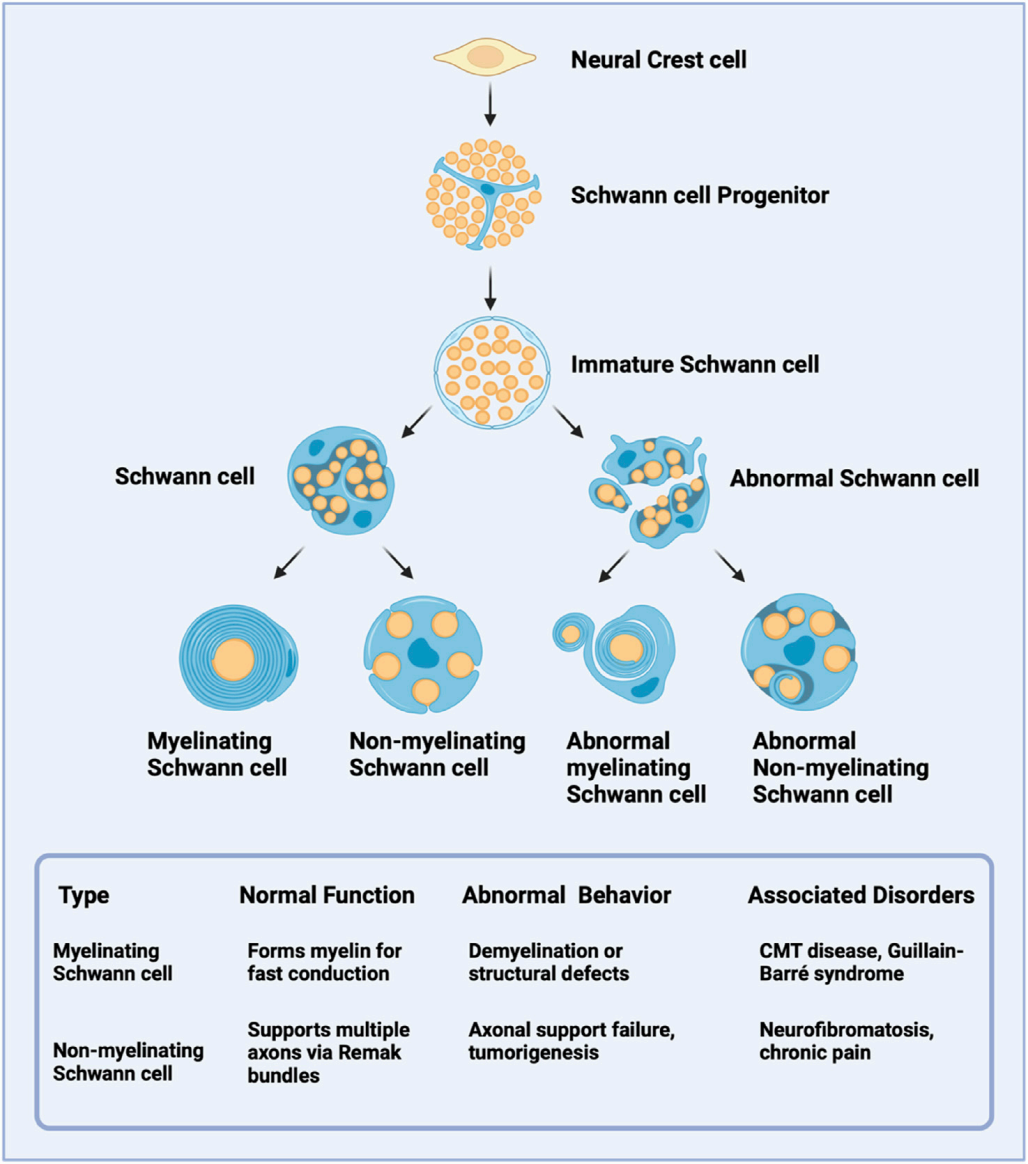
Developmental Lineage and Functional Characteristics of Schwann Cells. Schwann cells from neural crest-derived progenitors later highlight their differentiation into immature Schwann cells and subsequent maturation into myelinating or non-myelinating subtypes. Abnormal variants of each subtype are noted, along with their potential pathological implications.

Neurons and glia are the two main cell types that constitute the PNS. Neurons are specialized cells responsible for transmitting electrical and chemical signals, while glia serve as supportive cells. Astrocytes, oligodendrocytes, microglia, and Schwann cells are glial cells (von Bartheld et al., 2016) that participate in immune responses within the nervous system, provide structural support, maintain the extracellular environment, and regulate neurotransmitter levels (Grigorescu et al., 2024). Additionally, they are crucial for developing, maintaining, and repairing neural circuits (Allen and Lyons, 2018). Among glial cells, SCs are the most prominent in the PNS. They are vital for the growth, function, and regeneration of peripheral nerves (Gomez-Sanchez et al., 2015). In the mature nervous system, SCs are classified into two primary types: myelinating and non-myelinating (Figure 2). Myelinating SCs create the myelin sheath that encases all large-diameter peripheral axons, with each myelinating SC serving a single axon (Bolivar et al., 2020). In contrast, non-myelinating SCs wrap around multiple smaller axons, collectively encasing them within a structure known as a Remak bundle. Under normal physiological conditions, SCs can lead to PNS myelination, support axons, and regenerate damaged nerves (Jessen and Arthur-Farraj, 2019). Notably, Schwann cells exhibit remarkable plasticity functioning as multipotent progenitors capable of differentiating into various glial and non-glial cell types, including melanocytes, chondrocytes, etc (Taveggia and Feltri, 2022).

### 3.2 PNI facilitated by SCs in PDAC

In pathological conditions, SCs can influence cancer progression. Studies have shown that repair Schwann cells (rSCs), which arise in response to nerve regeneration after nerve damage caused by cancer cell invasion. These cells exhibit high plasticity and can generally differentiated from fully differentiated myelin cells, non-myelin cells (Remak), and terminal Schwann cells (Wei et al., 2024). Once converted to rSC, they facilitate the regeneration of damaged nerves. PNI, a hallmark of pancreatic ductal adenocarcinoma (PDAC), is present in nearly all cases and can manifest clinically as pain, paresthesia, numbness, or even paralysis in some patients (Homolova et al., 2024). Schwann cells have been identified as pro-tumorigenic cells within the tumor microenvironment, where they critically promote PNI (Cai et al., 2024). In human PDAC specimens, a direct association between SCs and cancer cells has been observed via electron microscopy and further validated by immunofluorescence (Sun C. et al., 2023; Su et al., 2020). Similar interactions have been reported in other maliganancies, including colorectal cancer (Han et al., 2022), thyroid cancer (Kandil et al., 2010), salivary duct carcinoma (Nakazato et al., 1985), and squamous cell carcinoma of the skin (Fahim et al., 2022), suggesting SCs may broadly regulate tumor innervation across cancer types (Azam and Pecot, 2016). Mechanistically, SCs secrete a variety of molecules to regulate tumorigenesis, while cancer cells and nerves reciprocally release signaling factors that facilitate PNI (Bakst and Wong, 2016). These findings highlight the complex interplay between different signalling molecules, neurotrophic factors, and chemokines in promoting PNI and tumor progression (Jiang et al., 2022). From a therapeutic perspective, further research is needed to determine whether broad targeting of SCs or specific inhibition of PNI-related molecular pathways would be more beneficial for patients. A deeper understanding of the cellular and molecular mechanisms driving perineural invasion could unveil novel opportunities for therapeutic intervention.

Recent studies have delineated the molecular signatures of sympathetic and sensory neurons innervating PDAC or healthy pancreas, revealing distinct transcriptomic patterns in tumor-associated neurons and their interactive networks with the TME. Further investigations demonstrate that pharmacological denervation induces a pro-inflammatory TME and enhances the efficacy of immune checkpoint inhibitors, while taxane-based drugs (e.g., nab-paclitaxel) suppress PDAC growth by inducing intratumoral neuropathy. Additionally, SCs play critical roles in cancer invasion, as tumor-activated Schwann cell tracks (TASTs) dynamically form migration pathways for cancer cells through c-Jun-dependent reprogramming, analogous to nerve repair processes. Spatial transcriptomic analyses reveal hypertrophic tumor-associated nerves in PDAC exhibit features of neural injury, including programmed cell death, Schwann cell proliferation signaling, and increased neuroglial cell turnover with concurrent apoptosis. These findings uncover the pathological mechanisms of nerve injury and repair within the tumor-nerve microenvironment, establishing novel therapeutic strategies targeting neural regulation in PDAC (Weitz et al., 2023; Deborde et al., 2022; Thiel et al., 2025).

### 3.3 SCs and other cell types work together to influence PNI

The abundance of SCs within the tumor is clinically significant, with higher SC densities typically correlating with increased PNI (Deborde et al., 2016). SCs recruit specific immune cells to enhance the PNI capability of tumor cells. Notably, SCs exhibit strong associations with myeloid-derived suppressor cells, regulatory T cells, and macrophages, implying potential crosstalk within the tumor microenvironment (Sun C. et al., 2023). Macrophages in the tumor microenvironment can polarize into distinct phenotypes, including M1, M2, and tumor-associated macrophages (TAMs) (Gao et al., 2022). M1 often eliminates the damaged cells in inflammatory tissue, while M2 often promotes the proliferation of neoplastic cells (Zhang and Sioud, 2023). TAMs further secrete factors (such as IL-8) to activate PDAC cells and promote PNI (Boutilier and Elsawa, 2021). SCs can secrete some factors that affect macrophages. Galectin-3 is secreted by SCs, which can induce a chemotactic response of the macrophage cells, and macrophage infiltration can induce peripheral neuropathy (Koyanagi et al., 2021). The reciprocal bFGF/IL-33 signaling axis between Schwann cells and TAMs also has found to play a critical role in facilitating perineural invasion (PNI) in pancreatic ductal adenocarcinoma (PDAC), creating a self-amplifying feedback loop that drives neural infiltration (Zhang et al., 2024). In addition, chemokine 2 (CXCL-2) and chemokine 1 (CXCL-1) secreted by SCs also promoted macrophage infiltration, which promotes pain perception (Zhang et al., 2023; Ntogwa et al., 2020). During nerve damage in cancer, SCs and macrophages engage in reciprocal interactions (De Logu et al., 2021). Activation of SCs TRPA1 stimulated NOX1-dependent H2O2 production to recruit macrophages. TRPA1 silencing reduces macrophage infiltration and alleviates mechanical pain in murine models (De Logu et al., 2017). Schwann cell-derived exosomes (SCDEs) suppress M1 macrophage polarization while stimulating M2 polarization, thereby diminishing inflammation, aiding in the regeneration of the myelin sheath and axons, and assisting in the repair of sciatic function (Ren et al., 2023; Sun J. et al., 2023; Fendl et al., 2023). In SCs, upregulating lncARAT facilitates axonal regeneration by attracting and activating macrophages (Yin et al., 2022).

Emerging evidence indicates that the interactions between cancer-associated fibroblasts (CAFs) and SCs significantly influence cancer progression in TME (Akkiz, 2023). CAFs, key stromal components in cancers like PDAC, drive tumor growth, invasion, and metastasis (Mao et al., 2021). SCs and CAFs can communicate through paracrine signaling, where they release signaling cytokines, chemokines, and other molecules. Such growth factors TGF-β can stimulate SCs to produce factors that promote tumor progression (Fang et al., 2023). SCs can facilitate the conversion of tumor cells and CAFs into more aggressive forms, such as basal-like tumor cells and inflammatory CAFs (iCAFs) (Wright et al., 2023). SCs boost the growth and movement of tumor cells via Midkine signaling. Additionally, they facilitate the conversion into iCAFs through the function of interleukin-1α (Xue et al., 2023). The presence of CAFs correlated significantly with PNI in breast cancer (Son et al., 2019). The PNI-associated transcript (PIAT) of the CAF can promote neural remodeling in pancreatic cancer by mediating the modification of m5C (Zheng et al., 2024).

**FIGURE 3:**
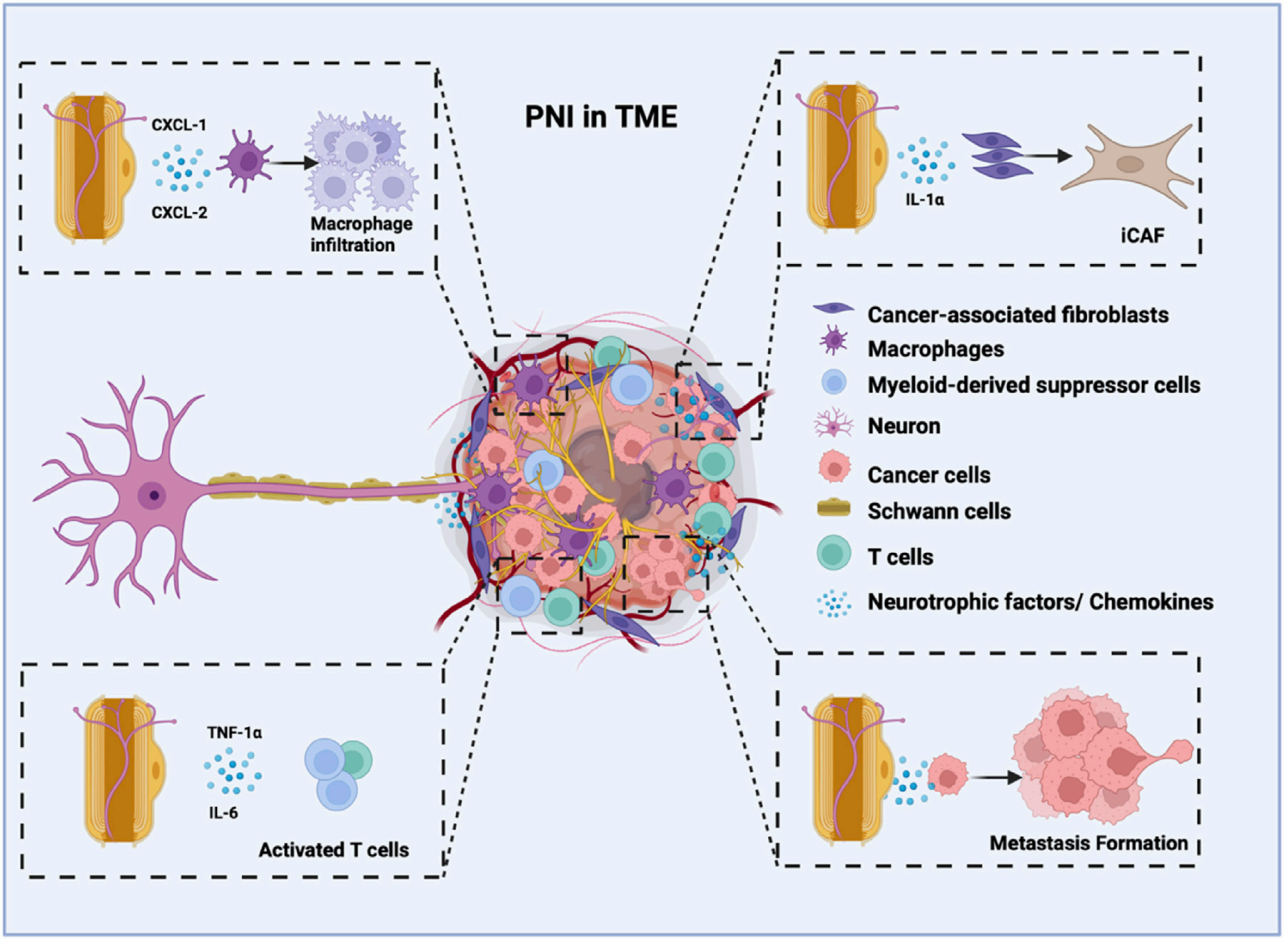
The role of Schwann cells in facilitating perineural invasion and tumor innervation, contributing to cancer metastasis. Schwann cells have a strong attraction to cancer cells. Cancer cells are attracted to Schwann cells or adapt to invade nerves, actively forming a tumour-nerve niche. Schwann cells can synergistically promote cancer metastasis by secreting neurotrophic factors and chemokines and by regulating various cells within the tumour microenvironment. This interaction creates a positive feedback loop between cancer cells and nerves.

In addition, SCs also recruit and polarize dendritic cells into regulatory dendritic cells (rDCs), which exhibit reduced pro-inflammatory cytokine expression and elevated anti-inflammatory markers (e.g., IL-10, TGF-β), further facilitating PNI (Schmidt et al., 2012; Troise et al., 2024). While the TME is known to mediate PNI through complex cellular interactions, the specific mechanisms by which SCs coordinate with other TME components to promote PNI in PDAC remain poorly characterized (Capodanno and Hirth, 2023). A comprehensive understanding of these interactions may not only clarify the biological underpinnings of PNI but also identify potential therapeutic targets (Figure 3).

## 4 Schwann cells molecular mediators promote perineural invasion in pancreatic ductal adenocarcinoma

### 4.1 Neurotrophins

**FIGURE 4:**
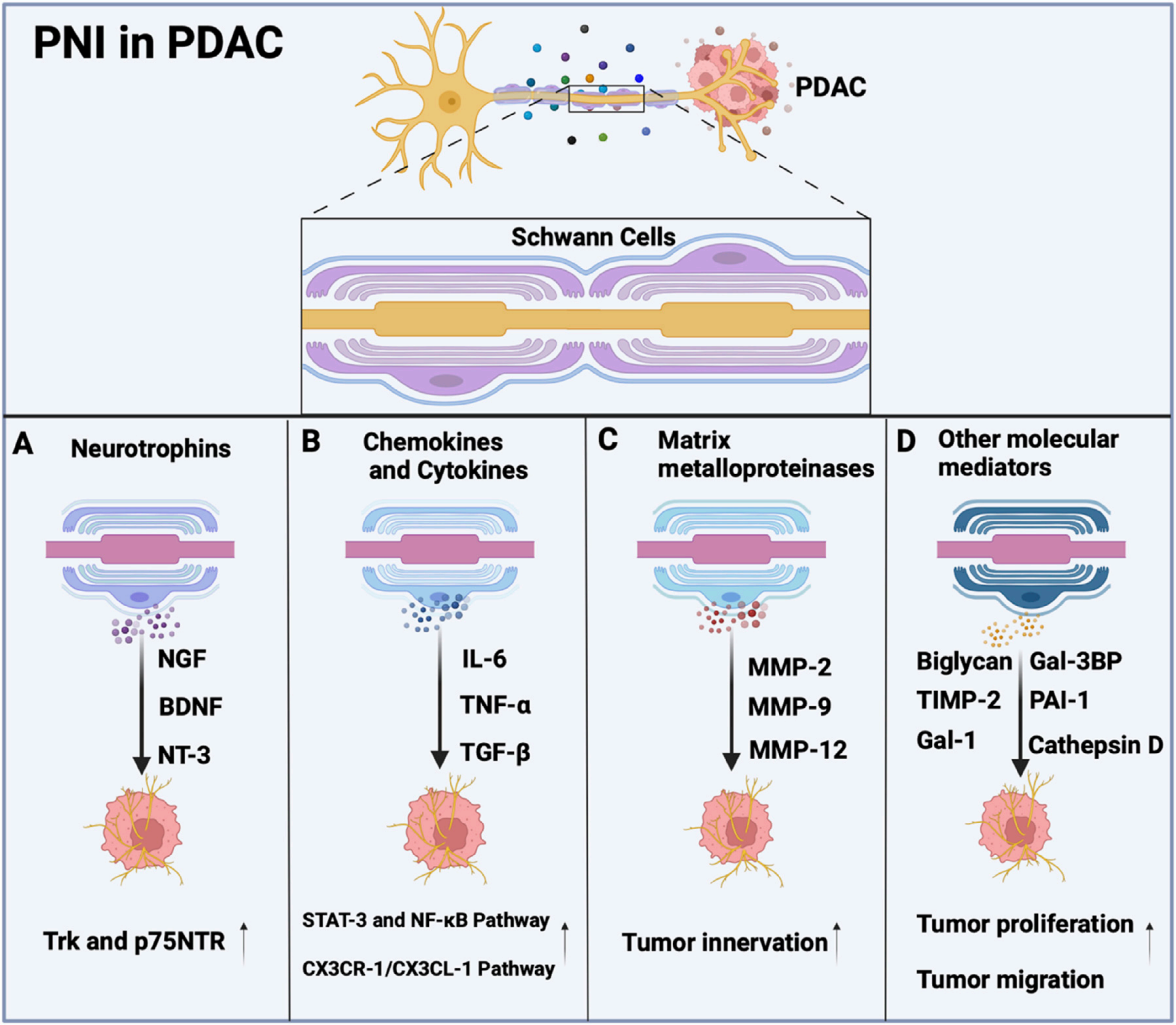
Molecular Mediators involved in Schwann cells. **(A)** Schwann cells secrete Neurotrophins, such as nerve growth factor (NGF), brain-derived neurotrophic factor (BDNF), and neurotrophin-3 (NT-3), along with their receptors (Trk and p75NTR) promote tumor cell invasion of nerves. **(B)** Schwann cells release cytokines such as IL-6, tumor necrosis factor-alpha (TNF-α), and TGF-β, which promote tumor cell survival, proliferation, invasion, and epithelial-mesenchymal transition (EMT) of pancreatic cancer cells through pathways involving STAT-3, NF-κB, or CX3CR-1/CX3CL-1. **(C)** MMP-2, MMP-9, and MMP-12 are secreted by Schwann cells, which is essential to exploring the role of SCs in tumor innervation. **(D)** Schwann cells release Gal-3BP, cathepsin D, PAI-1, biglycan, TIMP-2, and Gal-1 to promote tumor growth.

SCs play a direct role in regulating PDAC progression through the secretion of various signaling molecules, including neurotrophins (GDNF, NGF, BDNF, and NT-3), chemokines, cytokines, axonal guidance molecules, matrix metalloproteinases, and other molecular mediators (Figure 4). These neurotrophins and their receptors (Trk and p75NTR) mediate critical pathways in tumor development (Ferdoushi et al., 2020; Chang et al., 2019; Wang et al., 2014). Specifically, elevated nerve growth factor (NGF) enhances TrkA signalling to activate the MAPK pathway supporting critical processes for neuronal cell survival, differentiation, and axon development (Marlin and Li, 2015). GDNF promotes cancer cell migration by activating both PI3K/Akt and Ras-Raf-MEK-ERK signaling pathways (Veit et al., 2004). The BDNF/TrkB axis, along with NT-3/TrkC signaling, facilitates PDAC progression through perineural invasion, with TrkB being overexpressed in approximately 50% of PDAC cases compared to normal tissue (Sclabas et al., 2005; Akil et al., 2016). Importantly, blockade of NT-3/TrkC signaling has been shown to inhibit PNI in both pancreatic and prostate cancers (Li H. et al., 2019). The release of neurotrophins by SCs highlights their role in the intricate interaction between the PNS and tumor development, indicating their potential as targets for cancer therapy.

### 4.2 Chemokines and cytokines

Chemokines, including CXCL-12, CCL-2 and CCL-5, have been shown to recruit immune cells to the TME, which supports inflammation and enhances tumor progression (Demir et al., 2017; Huang et al., 2020; Aldinucci and Colombatti, 2014). SC-derived cytokines such as IL-6, TNF-α, and TGF-β play pivotal roles in promoting tumor cell proliferation, invasion, and perineural invasion (PNI) through activation of critical signaling pathways like STAT-3 and NF-κB (Bolin et al., 1995; Chu et al., 2020). A study showed that CCL21 and CXCL10 promote pancreatic cancer cell migration toward sensory neurons, and high CXCR3/CCR7 levels in PDAC patients correlate with increased cancer-associated pain (Hirth et al., 2020). Moreover, the CX3CR-1/CX3CL-1 signaling pathway has been implicated in the invasion of peripheral nerves and the spread of tumor cells along nerves both within and outside the pancreas (Marchesi et al., 2010). Similarly, the CXCL-12/CXCR-4 signaling pathway has been shown to enhance the invasiveness of prostate cancer cells in laboratory conditions and to elevate the number of nerves in living organisms (Song et al., 2024). SCs exhibit unique pro-tumorigenic behaviors by modulating pathways such as CXCL-5/CXCR-2/PI3K/AKT/GSK-3β/Snail-Twist to drive EMT and metastatic potential in lung cancer (Tian et al., 2022). Additionally, SCs co-cultured with tumor cells can secrete high levels of IL-6, which promotes the migration and invasion of the cancer cells through the activation of STAT-3 signaling, however, it can be mitigated by neutralizing IL-6 or inhibiting STAT-3 expression (Su et al., 2020). Furthermore, SCs-derived TGF-β contributes to the acquisition of aggressive properties by pancreatic cancer cells (Roger et al., 2019), while cancer-activated SCs form invasive tracks through the c-Jun-dependent mechanism (Arthur-Farraj et al., 2012). The release of chemokines and cytokines by SCs is crucial in cancer development, as it influences immune responses, fosters inflammation, and supports the survival and growth of tumor cells. Additional research is necessary to elucidate the precise mechanisms through which SCs-secreted chemokines and cytokines contribute to tumorigenesis, aiming to create innovative cancer treatment approaches.

### 4.3 Matrix metalloproteinases

Matrix metalloproteinases (MMPs) are crucial enzymes involved in extracellular matrix (ECM) remodeling, playing key roles in both physiological processes (e.g., nerve repair) and pathological conditions (e.g., cancer progression). In the PNS, SCs have been shown to release MMP-2 and MMP-9 (Okada et al., 2004; Okada et al., 2003), which facilitate ECM degradation to enable cellular migration and subsequent remyelination during nerve regeneration. Emerging evidence indicates that SC-derived MMPs also contribute to cancer pathogenesis by remodeling the tumor microenvironment (TME), thereby promoting cancer cell invasion and metastasis (Kessenbrock et al., 2010). Notably, MMP-9, an extracellular protease that is upregulated in SCs following peripheral nerve injury, plays a vital role in regulating axonal degeneration and recruiting macrophages to the injury site (Chattopadhyay et al., 2007). In addition, SCs might secrete MMP-2, cathepsin D, plasminogen activator inhibitor-1, or galectin-1, which collectively modify the TME to favor perineural invasion PNI (Ferdoushi et al., 2020). This phenomenon is particularly evident in cervical cancer, where cancer cell-activated SCs demonstrate increased MMP expression that degrades the ECM and creates a PNI-permissive microenvironment (Huang et al., 2020). Given the well-established role of SC-derived MMPs in nervous system development and regeneration, further investigation into their contribution to tumor innervation processes is warranted. Understanding these mechanisms may provide novel insights into the neural tropism of malignant cells and potential therapeutic targets.

### 4.4 Other molecular mediators

Proteomic analysis of the SC secretome identified multiple proteins involved in cell-cell adhesion, oxidation-reduction processes, and other functions. Key findings showed that proteins such as Gal-3BP, MMP-2, cathepsin D, PAI-1, biglycan, TIMP-2, and Gal-1 were upregulated in SC-conditioned medium. These proteins promoted pancreatic cancer cells proliferation and migration, and their effects were reversible by blocking antibodies (Ferdoushi et al., 2020). Notably, many of these proteins have been previously linked to pancreatic cancer progression. For example, blocking antibodies against Gal-3BP inhibited lung metastasis and prolonged survival in orthotopic pancreatic cancer mouse models (Choi et al., 2022). Downregulation of cathepsin D and Galectin-1 inhibits the migration of pancreatic cancer cells (Roda et al., 2009; Whiteman et al., 2007). The study identified several proteins that were not validated in this research, but these proteins have been confirmed to participate in the progression of other cancers and may likewise contribute to pancreatic cancer development. Additionally, depletion of the long noncoding RNA (lncRNA) plasmacytoma variant translocation 1 (PVT1) secreted by nonmyelinating Schwann cells inhibits tumor growth in PDAC (Sun C. et al., 2023). Understanding the molecules secreted by SCs and their mechanisms of action can provide a theoretical basis for developing therapeutic strategies to block PNI.

## 5 Conclusion and future perspectives

Schwann cells (SCs) play a pivotal role in the pathogenesis of perineural invasion (PNI) in pancreatic ductal adenocarcinoma (PDAC). During PNI, SCs dynamically interact with cancer cells and other components of the tumour microenvironment to facilitate nerve infiltration and metastasis. In addition, SCs contribute to neural remodelling and inflammatory responses by recruiting immune cells, thereby fostering an immunosuppressive niche that supports tumour progression. Neuro-immune interactions regulate the tumor immune microenvironment through multiple pathways, including adrenergic, cholinergic, and neuropeptide signaling. Preclinical and clinical data indicate that the sympathetic nervous system directly governs T cell fate via the adrenergic receptor ADRB1, driving their terminal differentiation into exhausted states and thereby suppressing anti-tumor immunity (Globig et al., 2023). Notably, pancreatic cancer patients using β-blockers exhibit survival benefits, underscoring the critical role of neuro-immune crosstalk in immune regulation. This discovery highlights a promising research direction for understanding and manipulating neuro-immune interactions in cancer therapy. SCs involvement in cancer-associated pain underscores their dual role in PDAC pathophysiology, as SC-nerve interactions amplify nociceptive signalling through cytokine release and neuronal sensitisation. What’s more, emerging evidence suggests that SC-derived exosomes can modulate the tumor microenvironment by influencing the behavior of various stromal and immune cells, such as CAFs and tumor-infiltrating immune cells. These exosomes may facilitate intercellular communication, reprogramming immune responses, promoting fibroblast activation, or even enhancing tumor cell invasiveness (Hao et al., 2023; Wong et al., 2022; Wei et al., 2019). Although there are currently no reports on the role of Schwann cell (SC)-derived exosomes in regulating pancreatic cancer progression, investigating the impact of SC-secreted exosomes on tumor cells holds significant scientific and clinical relevance.

Targeting Schwann cells is a promising therapeutic approach because disrupting the communication between SCs and cancer cells has the potential to inhibit critical processes such as PNI and metastasis. By disrupting the signalling pathways that facilitate this interaction, it may be possible to slow tumour progression, reduce the likelihood of cancer spreading to adjacent tissues and distant organs, and enhance the body’s immune response against the tumour, potentially leading to improved patient outcomes. In addition, modulating the tumour-promoting secretory profile of SCs could alter the tumour microenvironment in a way that reduces the supportive role of SCs in cancer progression. Targeting Schwann cells could be a valuable addition to existing therapies that not only aim to alleviate the pain associated with tumour invasion, but also aim to improve overall survival rates for patients battling PDAC. Further research is needed to uncover chemotherapy-induced peripheral neuropathy underlying mechanisms, assess whether chemotherapy-induced nerve damage fuels tumor progression, and develop better diagnostic tools to distinguish systemic from tumor-associated neuropathy.

Despite these promising avenues, significant challenges remain to fully elucidate the heterogeneity of Schwann cells and their context-dependent functions within the PDAC microenvironment. The function of SCs can vary significantly depending on their local environment and the stage of tumour development. This complexity complicates the development of targeted therapies, so addressing these knowledge gaps is critical to advancing SC-focused interventions into clinical practice. The use of advanced experimental models, including *in vitro* and *in vivo* systems that accurately mimic the tumour microenvironment, will be essential to gain a deeper understanding of SC biology in PDAC. In addition, detailed mechanistic studies will help elucidate the specific roles that SCs play in tumour progression and their interactions with cancer cells. By overcoming these challenges, researchers can pave the way for innovative therapeutic strategies that exploit the unique properties of Schwann cells, ultimately improving patient outcomes and offering new hope in the fight against pancreatic cancer.

## Glossary

SCs: Schwann cells
PNI: perineural invasion
PDAC: pancreatic ductal adenocarcinoma
TME: tumor microenvironment
PNS: peripheral nervous system
CT: Computed tomography
MR: Magnetic Resonance
CP: chronic pancreatitis
rSCs: repair Schwann cells
Remak: non-myelin Schwann cells
TAMs: tumor-associated macrophages
IL-8: interleukin-8
CXCL-1: chemokine 1
CXCL-2: chemokine 2
CXCL-5: chemokine 5
CXCL-12: chemokine 12
TRPA1: transient receptor potential ankyrin 1
NOX1: NADPH oxidase 1
CAFs: cancer-associated fibroblasts
TGF-β: Transforming growth factor-β
iCAFs: inflammatory CAFs
IL-6: Interleukin-6
IL-10: Interleukin-10
NGF: nerve growth factor
GDNF: glial cell-derived neurotrophic factor
BDNF: brain-derived neurotrophic factor
NT-3: neurotrophin-3
**p75** ^**NTR**^: p75 Neurotrophin Receptor
Trk: Tyrosine kinase receptor
TrkA: Tyrosine kinase receptor A
TrkB: Tyrosine kinase receptor B
TrkC: Tyrosine kinase receptor C
MAPK: Mitogen-activated protein kinase
PI3K: Phosphoinositide 3-kinases
Akt: protein kinase B
MEK: mitogen-activated protein kinase
ERK: extracellular-signal-regulated kinase
CCL-2: chemokine (C-C motif) ligand 2
CCL-5: chemokine (C-C motif) ligand 5
TNF-α: tumor necrosis factor-alpha
STAT-3: Signal transducer and activator of transcription 3
NF-κB: Nuclear factor kappa B
CX3CR-1: CX3C motif chemokine receptor 1
CX3CL-1: chemokine (C-X3-C motif) ligand 1
CXCR-2: C-X-C chemokine receptor type 2
CXCR-4: C-X-C chemokine receptor type 4
GSK-3β: Glycogen Synthase Kinase 3beta
EMT: epithelial-mesenchymal transition
MMPs: matrix metalloproteinase
MMP2: matrix metallopeptidase 2
MMP9: matrix metallopeptidase 9
MMP12: matrix metallopeptidase 12
Ach: acetylcholine
NE: norepinephrine
β-AR: β-adrenergic receptor
Gal-3BP: galectin-3–binding protein
PAI-1: plasminogen activator inhibitor-1 biglycan
TIMP-2: tissue inhibitor of metalloproteinases-2
Gal-1: galectin-1.
